# Comparison of accidental pediatric scald burns in a tertiary care center: hot cauldron burns versus accidental spill burns

**DOI:** 10.12688/f1000research.73840.1

**Published:** 2021-10-26

**Authors:** Kiran Kishor Nakarmi, Bishnu Deep Pathak, Dhan Shrestha, Pravash Budhathoki, Shankar Man Rai

**Affiliations:** 1 Department of Burns, Plastic and Reconstructive Surgery, Kirtipur Hospital, phect-NEPAL, Kathmandu, Bagmati Province, 44600, Nepal; 2 Department of Internal Medicine, Nepalese Army Institute of Health Sciences, College of Medicine, Kathmandu, Bagmati Province, 44600, Nepal; 3 Department of Internal Medicine, Mount Sinai Hospital, Chicago, Illinois, USA; 4 Department of Internal Medicine, Bronx-Lebanon Hospital, Bronxville, New York, USA

**Keywords:** accidental, pediatric, burns, immersion

## Abstract

**Background:** Scald burns result from exposure to high-temperature fluids and are more common in the pediatric age group. They occur mainly by two mechanisms: (i) spill and (ii) immersion (hot cauldron) burns. These two patterns differ in clinical characteristics and outcomes. Scalds cause significant morbidity and mortality in children. The objective of this study was to compare accidental spill burns and hot cauldron burns in a hospital setting.

**Methods:** An analytical cross-sectional study was conducted by reviewing the secondary data of scald cases admitted during the years 2019 and 2020 in a burn-dedicated tertiary care center. Total population sampling was adopted. Data analysis was done partly using SPSS, version-23, and Stata-15. Mann Whitney U-test and Chi-square/Fisher's exact test were done appropriately to find associations between different variables. Regression analysis was performed taking mortality events as the outcome of interest.

**Results:** Out of 108 scald cases, 43 (39.8%) had hot cauldron burns and 65 (60.2%) had accidental spill burns. Overall mortality was 16 (14.8%), out of which hot cauldron burns and accidental spill burns comprised 12 (75.0%) and 4 (25.0%), respectively. Multinomial logistic regression analysis showed the type of scald, age, and Baux score found to be associated with mortality. Every one-year increment in age had a 29% lower odds of occurrence of mortality event (adjusted odds ratio [OR], 0.71; 95% confidence interval [CI], 0.50-0.99, p=0.042). Likewise, every one-point increment in Baux score was associated with 19% higher odds of mortality (adjusted OR, 1.190; 95% CI, 1.08-1.32; p<0.001).

**Conclusions:** Accidental spill burns were more common but mortality was significantly higher for hot cauldron burns. The majority of burn injuries occurred inside the kitchen emphasizing appropriate parental precautions. The risk of mortality was significantly higher in burn events occurring outside the house, and burns involving back, buttocks, perineum, and lower extremities.

## Introduction

Scald burns are the most common types of burn injuries in children throughout the world. ^1^^,^^2^ These injuries are the leading cause of morbidity and mortality in the pediatric population. ^3^^,^^4^ They have long-term physical, psychological and economic impacts on the patients and their families. The majority of scald injuries occur at home and most of them are accidental and preventable. ^5^^–^^7^

Scalds result from exposure to hot liquids or steam. They occur mainly by two mechanisms: (i) spill and (ii) immersion burns. ^8^^–^^10^ Accidental spill burns are due to spillage of hot liquid such as boiling water, tea, milk, oil, soup, etc. These burns usually happen if a child gets in the way of an adult carrying hot fluids or accidentally play with the utensils filled with hot liquid and spill over their body. Immersion burns take place when children fall or put their hands/feet into a vessel containing hot liquid such as bathtub, tea/coffee pots, hot cauldrons, etc. ^3^^,^^6^^,^^11^^–^^14^ Past studies have shown that the patterns of these two scalds differ widely for different age groups, gender, and body parts involved, with differences in hospital stay and outcomes secondary to extent of burns. ^6^^,^^15^^–^^17^

The study of different types of scalds can help to control or reduce the predisposing factors and ultimately help in formulating plans and strategies for prevention. This study aimed to compare accidental spill and immersion (hot cauldron) scald burns in a tertiary care center and assess morbidity and mortality caused by them.

## Methods

### Study setting

The study was conducted in the tertiary care center Kirtipur Hospital, Kathmandu, Nepal. It is a national referral center for the management of burn injuries. This hospital has 100 beds in total. Out of these, 32 beds are allocated for burn cases only; of which eight are in Burn Intensive Care Unit (BICU). It takes care of the whole spectrum of burn cases from acute care to the full range of reconstructive services and rehabilitation.

### Study description and variables

This was a single-center, analytical, cross-sectional study carried out by reviewing the secondary data of scald burn cases admitted during two years (2019 and 2020). Out of all scalds, only pediatric cases (age < 18 years) were included in our study. Secondary data was collected from hospital records maintained in electronic form which included all of the acute scald cases admitted to the Plastic Surgery Ward and BICU. Scald cases managed on an outpatient basis were excluded from the study. Total population sampling was used. Those cases with incomplete records were excluded.

The data extracted from the patients' electronic records and stored in our database included demographic information like age, gender, and address of residence. All the immersion injury cases presented to our center had occurred due to falling into cauldrons filled with boiling water containing shredded straw and husk to feed cattle, which is commonly used in Nepal. Spill burns occurred due to spillage of hot liquids (boiling water, tea, coffee, milk, and oil). The mechanism of scalds was categorized into two broad groups: hot cauldron burns (HCB) and accidental spill burns (ASB). Data related to burn injury included type (mechanisms) of scalds, place of burn, pre-hospital intravenous fluid use, presence of infection at presentation, total body surface area, and body parts involved. Likewise, data of in-hospital interventions such as blood transfusion, escharotomy, necrosectomy, tangential excision, second excision, use of graft and/or flap, amputation were also taken. Outcome variables like duration of hospital stay and mortality were also noted.

Since we were collecting data from patients’ electronic records, there was possibility that the hospital staffs might have made errors during data entry in the records. This could give rise to information bias in our study. To reduce it to minimum, we cross-checked every information with patients’ admission files stored in hospital administratin section itself. Next, all the eligible cases were included in the study. So, there was minimal chance of selection bias as well.

### Ethical considerations

Ethical approval was taken from the Institutional Review Committee (IRC no. 006-2021), phect-NEPAL. Before conducting the study, permission was taken from the Department of Burns, Plastic and Reconstructive Surgery. The anonymity of patient information was well-maintained and thus patient consent was waived by the ethical review board.

### Statistics

Shapiro-Wilk W test showed that our continuous variables were distributed non-normally, so median and inter-quartile range (IQR) were calculated. Among categorical variables, Chi-square/Fisher’s exact test was applied to check the association between independent and dependent variables. Frequency and percentages were presented appropriately in tables. The level of significance was taken as p < 0.05, with a 95% confidence interval (CI) considering a 5% standard error. The analysis was run partly using IBM SPSS version 23, and Stata version 15.

*Logistic regression*

The dependent variable was mortality outcome, while the rest of the other variables affecting mortality were independent variables. For regression analysis, the outcome of interest as mortality event was taken and those who died in course of treatment were labeled as 1, and those who survived were labeled as 0. Initially binary logistic regression was run to see the effect of individual variables in mortality outcome. Only those variables which were found to be associated in Chi-square/Fisher’s exact test and significant continuous variables (age, length of hospital stay, and Baux score) were taken for logistic regression analysis. Later, multinomial logistic regression analysis was performed to check the exact effect of independent variables adjusting to the rest of the variables to check and nullify the confounding effect of different variables evaluated. Unadjusted odds ratio (OR) for binary logistic regression and adjusted OR for multinomial logistic regression analysis were presented with 95% CIs. The pseudo R ^2^ value for multinomial logistic regression analysis was 0.5367, indicating that our model predicts a similar outcome in about 54% of observations. Multicollinearity across the studied variables was automatically tested by STATA software and no observed variables were omitted across selected variables for multinomial logistic regression analysis.

## Results

A total of 120 pediatric patients with scald burns were admitted during the two-year period. Out of them, 12 were excluded from our study due to their incomplete records. Finally, 108 cases were analyzed. ^23^ Demographic and clinical characteristics of the patients are shown in Table 1. The median age of the patients was 2.8 (IQR = 1.6-4.0) years. In total, 61 (56.5%) of them were male and 47 (43.5%) were female. The majority of the burn injuries occurred in the kitchen (57.4%) and most of the patients were from the Low mountain region (60.2%) of Nepal.

**Table 1.** Cross-tabulation of different variables with the type of scalds.

SN	Characteristics	Total n(%)	HCB n(%)	ASB n(%)	p-value
1.	N	108(100.0)	43(39.8)	65(60.2)	
2.	Age (in years)	2.8(1.6-4.0)	3(2.0-4.0)	2.5(1.3-4.0)	0.362
3.	Sex (males)	61(100.0)	25(41.0)	36(59.0)	0.777
4.	Place of Burns:				0.152
	Kitchen	62(100.0)	21(33.9)	41(66.1)	
	Inside house	41(100.0)	21(51.2)	20(48.8)	
	Outside	5(100.0)	1(20.0)	4(80.0)	
5.	Baux Score	17.0(11.6-25.8)	17.5(14.0-27.0)	16.0(10.5-23.5)	0.307
6.	Total Body Surface Area (TBSA)	15.0(8.0-20.0)	15.0(8.0-22.0)	15.0(8.0-19.0)	0.463
7.	Body parts involved:				
	Head and Neck	22(100.0)	6 (27.3)	16 (72.7)	0.178
	Face	38(100.0)	11(28.9)	27(71.1)	0.089
	Chest	40(100.0)	15(37.5)	25(62.5)	0.706
	Abdomen	36(100.0)	15(41.7)	21(58.3)	0.781
	Back	31(100.0)	14(45.2)	17(54.8)	0.471
	Buttock	29 (100.0)	15(51.7)	14(48.3)	0.126
	Perineum	9(100.0)	5(55.6)	4(44.4)	0.479
	Upper Extremities	53(100.0)	20(37.7)	33(62.3)	0.665
	Hands	44(100.0)	16(36.4)	28(63.6)	0.544
	Lower Extremities	60(100.0)	24(40.0)	36(60.0)	0.965
8.	Pre-hospital Intravenous Fluid Use	22(100.0)	12(54.5)	10(45.5)	0.114
9.	In-hospital Blood Transfusion	28(100.0)	16(57.1)	12(42.9)	**0.030**
10.	Duration of hospital stay (days)	9.0(5.0-14.0)	9.0(5.0-13.0)	9.0(3.0-14.0)	0.237
11.	Infection at admission	13(100.0)	7(53.8)	6(46.2)	0.270
12.	Escharotomy	6(100.0)	3(50.0)	3(50.0)	0.681
13.	Necrosectomy	2(100.0)	1(50.0)	1(50.0)	1.000
14.	Tangential Excision	52(100.0)	18(34.6)	34(65.4)	0.287
15.	Second Excision	8(100.0)	4(50.0)	4(50.0)	0.710
16.	Graft use	50(100.0)	16(32.0)	34(68.0)	0.123
17.	Graft type				0.109
	Autograft	48(100.0)	15(31.3)	33(68.8)	
	Allograft	1(100.0)	0	1(100.0)	
	Both auto- and allografts	1(100.0)	1(100.0)	0	
17.	Amputation	2(100.0)	2(100.0)	0	0.156
18.	Use of flap	2(100.0)	2(100.0)	0	0.156
19.	In-hospital mortality	16(100.0)	12(75.0)	4(25.0)	**0.002**

Note: Continuous variables are expressed as median (IQR) and categorical variables as number (percentage). The p-value is derived from Mann Whitney U test for continuous variables and Chi-square/Fisher's exact test for categorical variables. SN, Serial Number; HCB, Hot Cauldron Burns; ASB, Accidental Spill Burns; TBSA, Total Body Surface Area.

Overall, 43 (39.8%) had HCB and 65 (60.2%) had ASB. There was no significant difference in age, gender, place of burns, and pre-hospital intravenous fluid use in the two groups of scald patients. Baux score was higher in HCB (17.5, IQR = 14.0-27.0) but the median body surface area of burns was equal (15%) in both the groups. In total, 13 cases (12.0%) had an infection at presentation, which was higher in HCB (53.8%) than in ASB (46.2%). Neither of them were statistically significant.

The most commonly involved body parts were lower extremities (55.6%) followed by upper extremities (49.1%) and hands (40.7%). Of all the lower extremities burns, 36 (60.0%) were from ASB, and 24 (40.0%) were from HCB. None of these were statistically significant.

Tangential excision was performed in 52 (48.1%) patients, of which 34 (65.4%) and 18 (34.6%) belonged to ASB and HCB groups, respectively. Cases requiring the second excision (7.4%) were equally distributed in both groups (50.0%). Only two patients required flap and amputation and they were from the HCB group.

Overall mortality was 16 (14.8%). Out of total deaths, 12 (75%) were from HCB and four (25%) from the ASB group. This was statistically significant (p = 0.002). Requirement of in-hospital blood transfusion was significantly higher (p = 0.030) in HCB (57.1%) than ASB cases (42.9%). The median duration of hospital stay in both groups was nine days (IQR in HCB: 5.0-13.0, ASB: 3.0-14.0).

A significant association was seen between the dependent variable mortality and type of scalds (p = 0.002), place where burn injury took place (p = 0.010), and involvement of body parts like back (p = 0.015), buttock (0.001), perineum (p = 0.026) and lower extremities (0.005) ( Table 2).

**Table 2.** Cross-tabulation of different variables with mortality outcome using Chi-square/Fisher’s exact test.

Variables		Total	Mortality n(%)		p-value
			No	Yes	
Gender	Male	61(100.0)	51(83.6)	10(16.4)	0.599
	Female	47(100.0)	41(87.2)	6(12.8)	
Type of Scalds	HCB	43(100.0)	31(72.1)	12(27.9)	**0.002**
	ASB	65(100.0)	61(93.8)	4(6.2)	
Place of burn injury	Outside	5(100.0)	3(60.0)	2(40.0)	**0.010**
	Home	41(100.0)	31(75.6)	10(24.4)	
	Kitchen	62(100.0)	58(93.5)	4(6.5)	
Head and Neck	Yes	22(100.0)	18(81.8)	4(18.2)	0.737
	No	86(100.0)	74(86.0)	12(14.0)	
Face	Yes	38(100.0)	33(86.8)	5(13.2)	0.721
	No	70(100.0)	59(84.3)	11(15.7)	
Chest	Yes	40(100.0)	34(85.0)	6(15.0)	0.967
	No	68(100.0)	58(85.3)	10(14.7)	
Back	Yes	31(100.0)	22(71.0)	9(29.0)	**0.015**
	No	77(100.0)	70(90.9)	7(9.1)	
Abdomen	Yes	36(100.0)	28(77.8)	8(22.2)	0.125
	No	72(100.0)	64(88.9)	8(11.1)	
Buttock	Yes	29(100.0)	19(65.5)	10(34.5)	**0.001**
	No	79(100.0)	73(92.4)	6(7.6)	
Perineum	Yes	9(100.0)	5(55.6)	4(44.4)	**0.026**
	No	99(100.0)	87(87.9)	12(12.1)	
Upper Extremities	Yes	53(100.0)	46(86.8)	7(13.2)	0.644
	No	55(100.0)	46(83.6)	9(16.4)	
Hands	Yes	44(100.0)	38(86.4)	6(13.6)	0.775
	No	64(100.0)	54(84.4)	10(15.6)	
Lower Extremities	Yes	60(100.0)	46(76.7)	14(23.3)	**0.005**
	No	48(100.0)	46(95.8)	2(4.2)	
Pre-hospital intravenous fluid	Yes	22(100.0)	19(86.4)	3(13.6)	1
	No	86(100.0)	73(84.9)	13(15.1)	
In-hospital Blood transfusion	Yes	28(100.0)	23(82.1)	5(17.9)	0.555
	No	80(100.0)	69(86.3)	11(13.8)	
Infection at admission	Yes	13(100.0)	11(84.6)	2(15.4)	1
	No	95(100.0)	81(85.3)	14(14.7)	
Tangential Excision	Yes	52(100.0)	46(88.5)	6(11.5)	0.356
	No	56(100.0)	46(82.1)	10(17.9)	
Escharotomy	Yes	6(100.0)	5(83.3)	1(16.7)	1
	No	102(100.0)	87(85.3)	15(14.7)	
Graft (yes or no)	Yes	50(100.0)	44(88.0)	6(12.0)	0.445
	No	58(100.0)	48(82.8)	10(17.2)	
Graft Type	Autograft	48(100.0)	43(89.6)	5(10.4)	0.145
	Allograft	1(100.0)	1(100.0)	0(0.0)	
	Auto and Allo	1(100.0)	0(0.0)	1(100.0)	
Flap	Yes	2(100.0)	2(100.0)	0(0.0)	1
	No	106(100.0)	90(84.9)	16(15.1)	
Second excision	Yes	8(100.0)	7(87.5)	1(12.5)	1
	No	100(100.0)	85(85.0)	15(15.0)	
Amputation	Yes	2(100.0)	2(100.0)	0(0.0)	1
	No	106(100.0)	90(84.9)	16(15.1)	
Necrosectomy	Yes	2(100.0)	1(50.0)	1(50.0)	0.276
	No	106(100.0)	91(85.8)	15(14.2)	

Note: All these categorical variables are expressed as number (percentage). The p-value is derived from Chi-square/Fisher’s exact test. HCB, Hot Cauldron Burns; ASB, Accidental Spill Burns.

### Logistic regression

Binary and multinomial logistic regression analysis was run to check association across variables showing significant association by Chi-square/Fisher’s exact test and continuous variables namely, patient’s age, length of hospital stay, and Baux score ( Table 3). Binary logistic regression showed higher mortality in HCB in comparison to ASB type of scald. Similarly, burns occurring outside the house had a higher association with mortality. Involvement of the back, buttock, perineum, and lower extremities was found to be associated with higher odds of mortality. However, adjusting independent variables and continuous variables (age, Baux score, length of stay) showed the only type of scald, age, and Baux score found to be associated with mortality. Every one-year increment in age has a 29.0% lower odds of occurrence of mortality event (adjusted OR, 0.71; 95% CI, 0.50-0.99, p = 0.042). Likewise, every one-point increment in Baux score was associated with 19% higher odds of mortality (adjusted OR, 1.19; 95% CI, 1.08-1.32; p < 0.001).

**Table 3.** Cross-tabulation of different variables with mortality outcome using multinomial logistic regression.

Mortality	uOR	[95% Conf. Interval]		z	P>|z|	aOR	[95% Conf. Interval]		z	P>|z|
Type (ASB®)										
HCB	5.903226	1.757944	19.8232	2.87	0.004*	40.93118	3.598128	465.6204	2.99	0.003*
Place of burn (Kitchen®)										
Outside	9.666666	1.23679	75.55403	2.16	0.031*	17.62378	.2270896	1367.732	1.29	0.196
Home	4.677419	1.355177	16.1442	2.44	0.015*	1.985822	.2886093	13.66376	0.70	0.486
Back (No®)										
Yes	4.090909	1.364786	12.26239	2.52	0.012*	.2862989	.030569	2.681378	−1.10	0.273
Buttock (No®)										
Yes	6.403509	2.066449	19.84318	3.22	0.001*	1.834235	.2428109	13.85612	0.59	0.557
Perineum (No®)										
Yes	5.8	1.365009	24.64453	2.38	0.017*	.4362471	.0299323	6.358065	−0.61	0.544
LE (No®)										
Yes	6.999993	1.505288	32.55184	2.48	0.013*	14.85796	.9267597	238.2052	1.91	0.057
Age	.9742711	.8173489	1.161321	−0.29	0.771	.7059931	.5048816	.9872142	−2.04	0.042*
Length of hospital stay	1.010032	.9505634	1.073222	0.32	0.747	.9490669	.8554489	1.05293	−0.99	0.324
Baux	1.099279	1.048855	1.152127	3.95	0.000*	1.194787	1.081358	1.320114	3.50	0.000*
Constant						.0001546	1.58e-06	.0151046	−3.75	0.000

Note: *, significant; ®, reference; uOR, unadjusted Odds ratio; aOR, adjusted Odds ratio; HCB, Hot Cauldron Burns; ASB, Accidental Spill Burns; LE, Lower Extremities.

## Discussion

In our study, HCB and ASB accounted for 39.8% and 60.2% of total cases respectively. Although there are numeric differences across the two groups in terms of demographic and clinical profile, statistical significance exists in mortality and in-hospital blood transfusion only. Overall mortality was 16 (14.8%), out of which 12 (75%) were from HCB and 4 (25%) from the ASB group. Blood transfusion was required more in HCB cases (57.1%) than in ASB cases (42.9%). Mortality was higher in male gender (16.4%), burns outside the house (40.0%), body parts involving head and neck (18.2%), chest (15.0%), back (29.0%), abdomen (22.2%), buttocks (34.5%), perineum (44.4%), lower extremities (23.3%) and those who underwent escharotomy (16.7%) and necrosectomy (50.0%). Out of these, burns outside house (p = 0.010), involvement of buttocks (p = 0.001), back (p = 0.015), perineum (p = 0.026) and lower extremities (p = 0.005) were statistically significant.

A study in India ^18^ reported the mortality in pediatric scald burns to be 3.1% which is lower compared to our number (14.8%). The plausible explanation for these differences is the difference in the site of study, sample size, and duration of the study. Another reason could be because our center is a national referral center for burns, so it is likely that more complicated and extensive cases are being referred here. Another study in Kashmir, India ^19^ showed mortality from scalds to be 10.7% which is comparable to our findings. The most common place of burn event in our study was a kitchen (57.4%) which is comparable to a study (64.7%) done by Riedlinger DI *et al.* Similarly, grafting was done in 41.2% of patients which also corresponds to our study (46.3%). ^5^ Another study ^6^ depicted the incidence of accidental immersion and spill burns as 5.4% and 81.4%, respectively, which differs from our findings where immersion (hot cauldron) and spill burns comprise 39.8% and 60.2%, respectively. Similarly, a Turkish study ^3^ showed that the most frequent cause of burn was scalding from spillage of hot water (59.7%) followed by bath scalding (i.e. immersion injury) accounting for 26% of cases. This is in line with the percentage of spill burns in our case. Likewise, in Japan, immersion burn (59.3%) was reported to be higher than spill burns (40.7%). ^16^ This could be due to the provision of the bathtub in developed countries like Japan where there is a high chance of children climbing up and falling into bathtubs.

The most commonly involved body parts were lower limbs (55.6%) and upper limbs (49.1%) in our study. This finding contradicts a study by Drago DA *et al*, ^6^ where the upper torso (25.3%) and upper limbs (24.1%) were maximally involved. A Japanese study ^16^ revealed that the most common sites of immersion injury were trunk and legs (80%) followed by arms, and those of spill burns were trunk (91.7%) followed by head/neck and arms. In our case, the most common body parts involved were lower followed by upper extremities. The same study showed the average body surface area of scald as 11.3% which is slightly lower than ours (15.0%). Immersion-related burns were more likely to be located on the lower half of the body involving buttocks, thighs, legs, and feet in a French study. ^17^ Another also found out that most scalds occurred on the upper limbs. ^20^ These discrepancies could be due to different sample sizes and sites of study. The mean total body surface area reported in a study from Arizona ^21^ was 8.0% which is much lower than ours (15.0%). The difference could be due to our center being a referral center where complicated burn cases are being referred from all over the country. However, the mean length of hospital stay in this study ^21^ (8 days) is similar to our study (9 days).

In a study from Ontario, ^15^ Children with spill burns had a shorter average length of hospital stay (10.8 days) compared to those involved in bathtub immersion burns (18.3 days). In our study, the median duration of hospital stay was equal in both the groups of scalds (nine days) though it was not statistically significant. Likewise, in the same study, the mean age in both scald groups was 1.8 years whereas, in our setting, the median age in HCB and ASB were 3.0 and 2.5 years, respectively. A study from Beijing ^22^ showed that the scald burns most commonly occurred in the kitchen which supports our result.

There are some limitations of this study that need to be mentioned. The number of cases was lower than expected because of the ongoing coronavirus disease 2019 (COVID-19) pandemic. Lack of complete data also excluded a significant portion of cases from the study. The data also fails to analyze the economic status of children which could make them susceptible not only to a certain type of scald burn but also limits the access to first aid and primary care, hence affecting the outcome. Moreover, this is a single-center study over a short period of time so, the results may not be applicable to the whole country. For that purpose, multi-center studies conducted over a longer duration are recommended.

## Conclusions

ASB was more common in our setting but mortality was higher in HCB. The latter were more likely to require an in-hospital blood transfusion. Most of the scalds occurred in the kitchen so parental precautions are necessary. There were no other significant differences between these two groups. Overall in-hospital deaths were higher than in other countries. The risk of mortality was significantly higher in burn events occurring outside, and those involving the back, buttocks, perineum, and lower extremities. So, special focus should be given to these factors during management.

## Data availability

Figshare: Comparison of Accidental Pediatric Scald Burns in a Tertiary Care Center: Hot Cauldron Burns versus Accidental Spill Burns.sav. http://doi.org/10.6084/m9.figshare.16583501. ^23^

Data are available under the terms of the Creative Commons Attribution 4.0 International license (CC-BY 4.0).
