# Supplementary material for: Comparison of accidental pediatric scald burns in a tertiary care center: hot cauldron burns versus accidental spill burns
Source: F1000Res. 2022 Jan 10;10:1086. Originally published 2021 Oct 26. [Version 2] doi: 10.12688/f1000research.73840.2 (PMC8758969; doi:10.12688/f1000research.73840.2)
Supplement: Peer review history

# Peer review: Reviewer response for version 2

**DOI:** 10.5256/f1000research.119819.r119126

**Published:** 2022-01-13

**Authors:** Saidur Rahman Mashreky

**Affiliations:** 1 Centre for Injury Prevention and Research Bangladesh, Dhaka, Bangladesh

Thank you for addressing the issues.

Is the work clearly and accurately presented and does it cite the current literature?

Yes

If applicable, is the statistical analysis and its interpretation appropriate?

Yes

Are all the source data underlying the results available to ensure full reproducibility?

Yes

Is the study design appropriate and is the work technically sound?

Partly

Are the conclusions drawn adequately supported by the results?

Partly

Are sufficient details of methods and analysis provided to allow replication by others?

Yes

Reviewer Expertise:

Injury Prevention including burns, Non Communicable disease including mental health

I confirm that I have read this submission and believe that I have an appropriate level of expertise to confirm that it is of an acceptable scientific standard.

# Peer review: Reviewer response for version 1

**DOI:** 10.5256/f1000research.77525.r100987

**Published:** 2021-12-21

**Authors:** Roshan Acharya

**Affiliations:** 1 Department of Internal Medicine, Cape Fear Valley Medical Center, Fayetteville, NC, USA

1. The authors have tried to compare the prevalence and outcome of hot cauldron burns versus accidental spill burns in a tertiary care hospital.
2. The authors have also explored various factors that are associated with mortality in burn patients in the hospital. 

1. The statistical methods are sound and the results are well presented.
2. The authors have well discussed the existing relevant literature. However, as the authors have rightly pointed out, the number of the patients included in the study seems to be low for the number of cases, but it is understandable being a single center study taking pediatric burn patients. Secondly, the prevalence of different types of burn cases may not be relevant in other settings.
3. Overall, the study has been well conducted and the manuscript has been well written according to the context.

Is the work clearly and accurately presented and does it cite the current literature?

Yes

If applicable, is the statistical analysis and its interpretation appropriate?

Yes

Are all the source data underlying the results available to ensure full reproducibility?

Yes

Is the study design appropriate and is the work technically sound?

Yes

Are the conclusions drawn adequately supported by the results?

Yes

Are sufficient details of methods and analysis provided to allow replication by others?

Yes

Reviewer Expertise:

Clinical, Critical Care, Pulmonology

I confirm that I have read this submission and believe that I have an appropriate level of expertise to confirm that it is of an acceptable scientific standard.

# Peer review: Reviewer response for version 1

**DOI:** 10.5256/f1000research.77525.r97945

**Published:** 2021-11-03

**Authors:** Saidur Rahman Mashreky

**Affiliations:** 1 Centre for Injury Prevention and Research Bangladesh, Dhaka, Bangladesh

Thank you for conducting an exciting study in the field of burn prevention.

 The study's title is 'Comparison of accidental pediatric scald burns in a tertiary care center: hot cauldron burns versus accidental spill burns'. The study aimed to compare accidental spill and immersion (hot cauldron) scald burns in a tertiary care center and assess morbidity and mortality caused by them.

 The objective of the study is less clear. However, in this study, the authors tried to determine the type of scald burn that is more prevalent and attempted to explore the association between the type of scald burn and the severity of morbidity and mortality. However, to answer this kind of research question, authors need to select an analytical study design. 

 The conclusion section of the article was not much aligned with your study title and objective.

 In my understanding, it is better to redesign the study to see the distribution of scald burn and its consequence.

Is the work clearly and accurately presented and does it cite the current literature?

Yes

If applicable, is the statistical analysis and its interpretation appropriate?

Yes

Are all the source data underlying the results available to ensure full reproducibility?

Yes

Is the study design appropriate and is the work technically sound?

Partly

Are the conclusions drawn adequately supported by the results?

Partly

Are sufficient details of methods and analysis provided to allow replication by others?

Yes

Reviewer Expertise:

Injury Prevention including burns, Non Communicable disease including mental health

I confirm that I have read this submission and believe that I have an appropriate level of expertise to confirm that it is of an acceptable scientific standard, however I have significant reservations, as outlined above.

# Response

**Published:** 2021-12-25

**Authors:** Bishnu Pathak

**Affiliations:** Nepalese Army Institute of Health Sciences College of Medicine, Nepal

Dear Sir,

 Thank you very much for reviewing our article and for providing healthy, constructive, and relevant comments. We owe you a great debt of gratitude for the same.

 We have tried to make the necessary corrections as suggested by you in the new version. There are a few things that we would like to put forward. 

 1. You have commented on the design of the study. Our study is an analytical cross-sectional study. We have done comparisons at two different levels. Firstly, between the types of burns (hot cauldron burns and accidental spill burns). Secondly, a comparison is done between survivors and non-survivors, though this is not mentioned in the title of the article. It is because our primary objective was to analyze the types of scald burns in children. But, during analysis, we found many factors interestingly affecting the mortality, due to which we did a second comparison as well. This could be helpful to the scientific literature that is lacking these sorts of findings.

 2. We have written the conclusion as suggested by you, such that it sounds being aligned with the tile of the study.

 3. We have also tried to write objectives more clear than before.

 We humbly request you to look upon these changes.

 Thank you

 Regards

 Bishnu Deep Pathak
